# Supplementary material for: Identification of compounds that repress DUX4 expression in facioscapulohumeral muscular dystrophy
Source: bioRxiv. 2026 Mar 17:2026.03.09.710626. Preprint. [Version 2] doi: 10.64898/2026.03.09.710626 (PMC13015533; doi:10.64898/2026.03.09.710626)
Supplement: Supplement 1 [file media-1.pdf]

Supplementary Figure S1

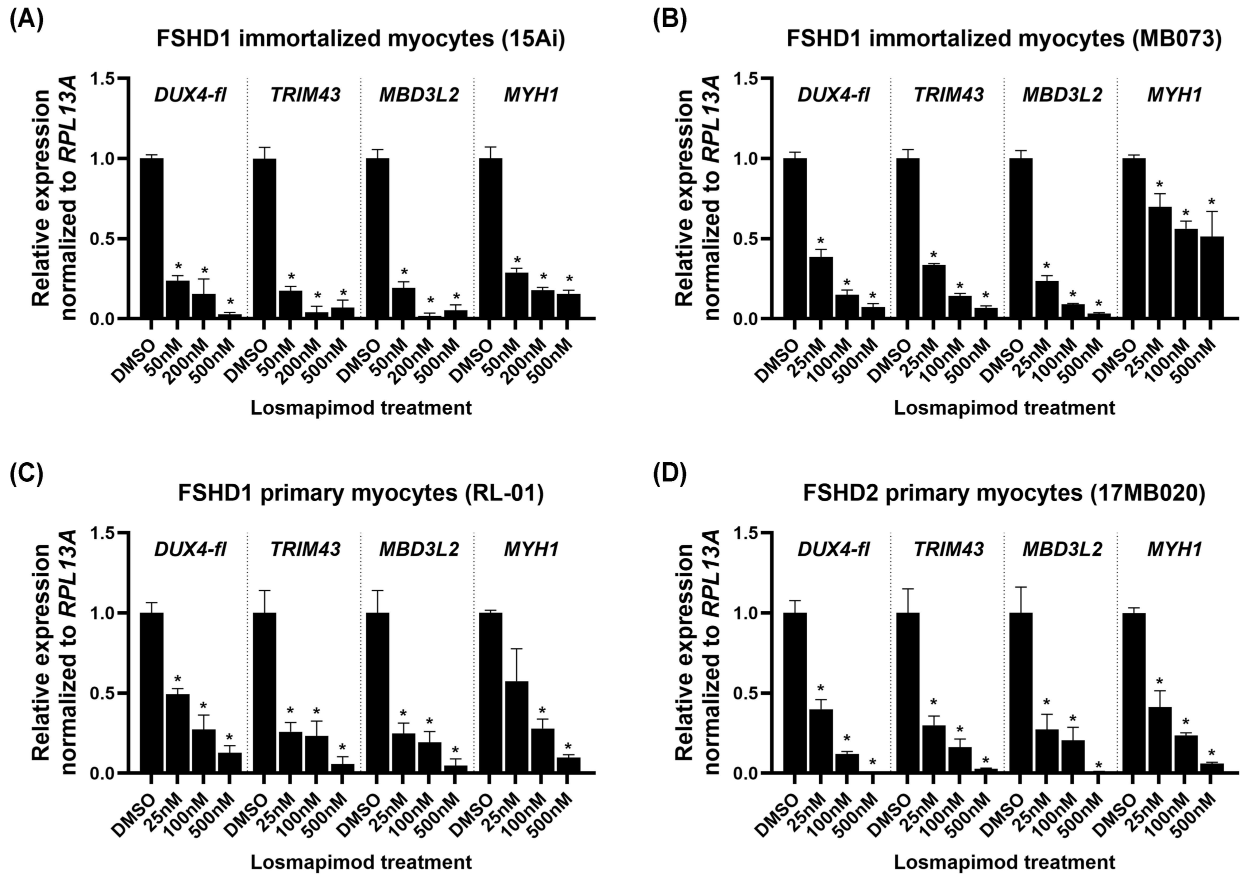

Supplementary Figure S1. Losmapimod treatment suppresses *DUX4-fl*, DUX4-FL targets, and *MYH1* in multiple FSHD muscle cell lines.

Two immortalized FSHD1 lines (A-B), one primary FSHD1 line (C), and one primary FSHD2 line (D) of patient myoblasts were differentiated and treated with losmapimod at 25 nM (or 50 nM for 15Ai cells), 100 nM, and 500 nM. Expression levels of *DUX4-fl,* DUX4-FL target genes *MBD3L2* and *TRIM43,* and the differentiation marker *MYH1* were assessed by RT-qPCR and normalized to *RPL13A*. Data are plotted as the mean ± standard error of the mean (SEM) of at least three independent experiments, with average expression of vehicle-treated cells (DMSO) set to 1. *$p < 0.05$ is from comparing to DMSO.

Supplementary Figure S2

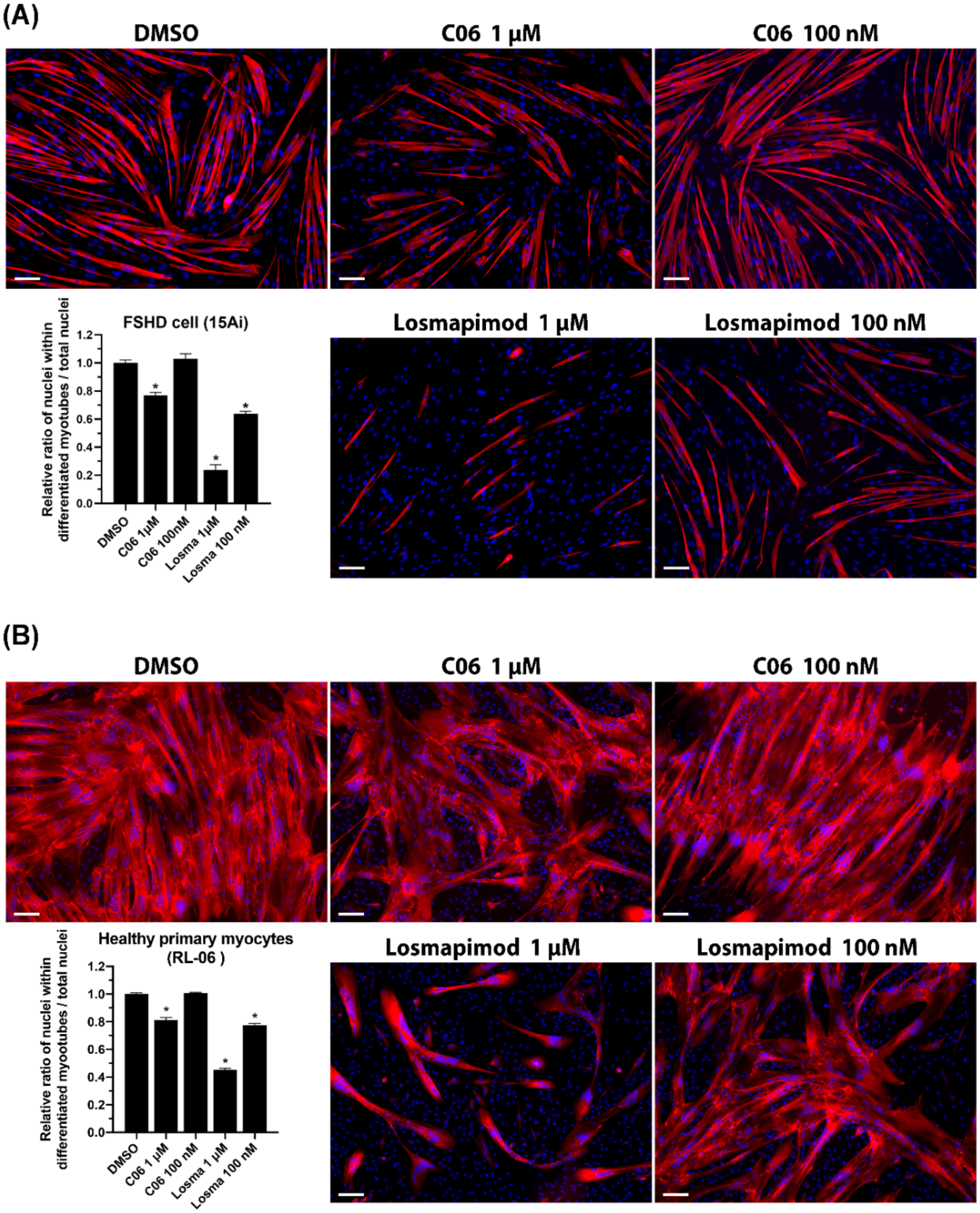

Supplementary Figure S2. Effects of C06 and losmapimod on myogenic differentiation in FSHD1 and healthy myocytes.

(A) Immunofluorescence staining of differentiated FSHD1 myocytes (15Ai) treated with DMSO, C06 (1 µM or 100 nM), or losmapimod (1 µM or 100 nM) for 4 days. MYH1 was detected by immunostaining (red), and nuclei were counterstained with DAPI (blue). **(B)** Immunofluorescence staining of differentiated healthy primary myocytes (RL-06) under the same treatment conditions. MYH1 (red) and DAPI (blue) staining are shown. Quantification of the ratio of nuclei within MYH1-positive areas is shown (mean ± SEM, *$p < 0.05$ compared with DMSO). Scale bar = 100 µm.

Supplementary Figure S3.

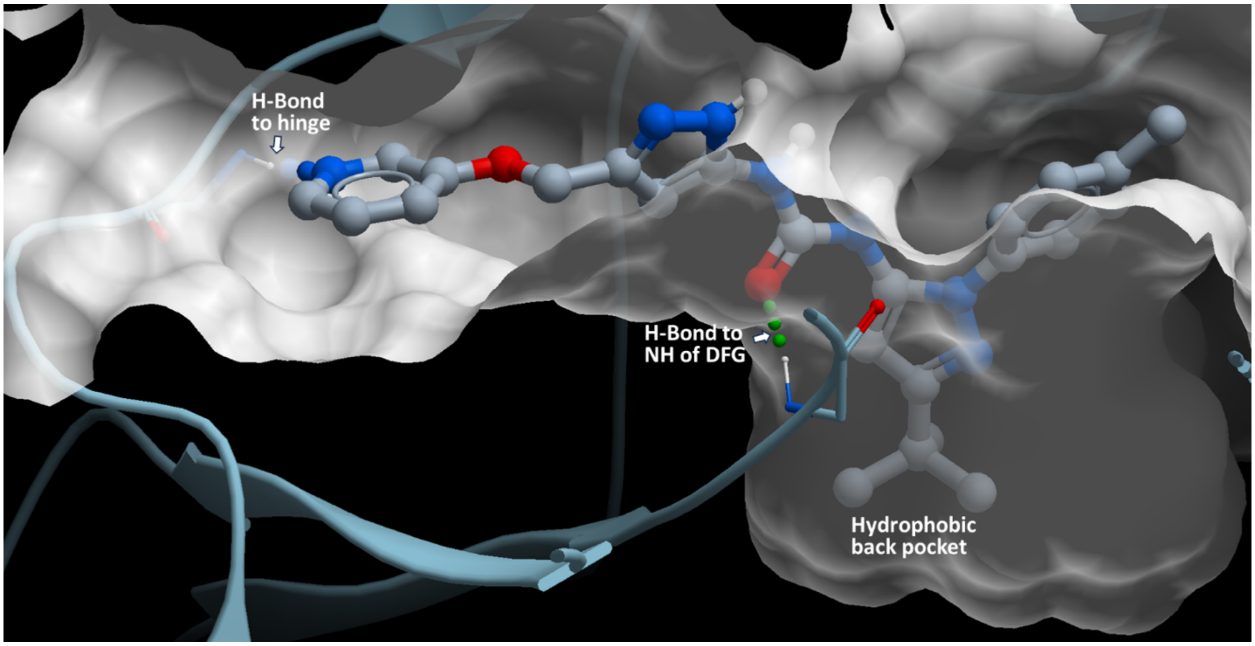

Supplementary Figure S3. Crystal structure of C06 bound to PYK2.

Crystal structure of a compound identical to C06 bound to the PYK2 non-receptor tyrosine kinase, reported by Han et al[1]. Interactions with the pyridine, carbonyl, and *tert*-butyl moieties of C06 are shown. The structure was rendered from the PDB file (PDB: 3FZT, resolution 2.00Å) reported by Han et al[1]. using the ICM Browser (Molsoft).

Supplementary Figure S4

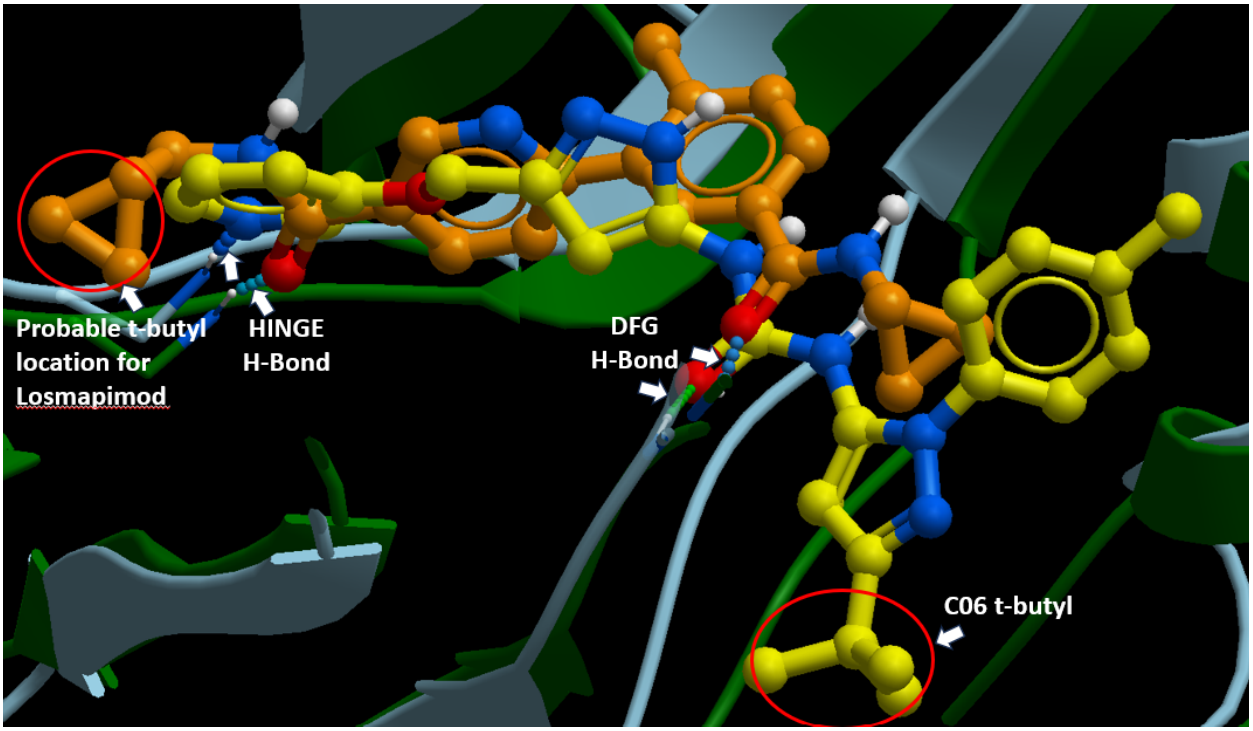

Supplementary Figure S4. Structural comparison of C06 and losmapimod binding modes.

Overlay of C06 (yellow; PDB: 3FZT) and a comparator crystal structure representing the binding mode of losmapimod (brown; PDB: 3IPH) in the kinase active site. The *tert*-butyl group of losmapimod is positioned near the mouth of the binding pocket in a solvent-exposed region, whereas the *tert*-butyl group of C06 extends into the deeper back pocket. Key hydrogen-bond (H-Bond) interactions with the hinge region and the DFG motif are indicated. Structural rendering was performed using ICM Browser (Molsoft).

Supplementary Figure S5

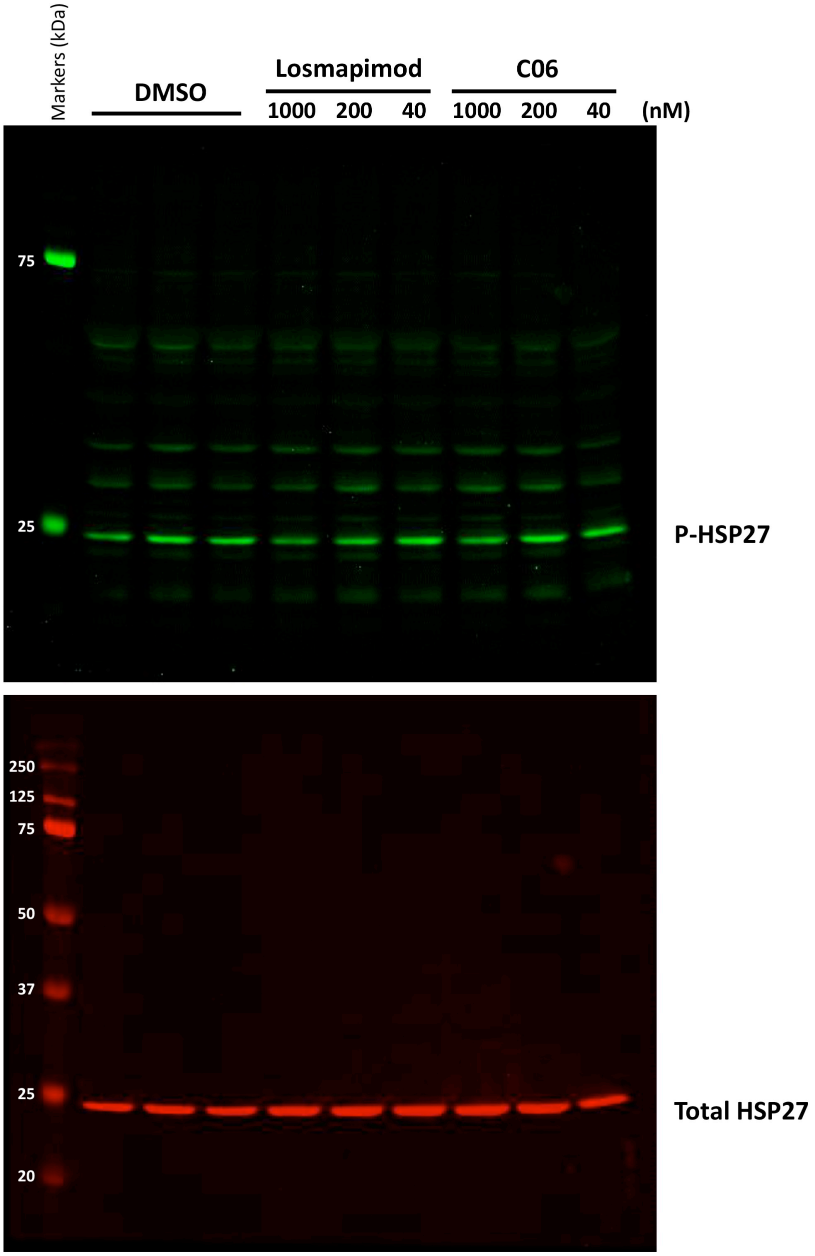

Supplementary Figure S5. Original western blots corresponding to Figure 7B.

Uncropped western blot images for phosphorylated HSP27 (P-HSP27) and total HSP27 corresponding to the data shown in Figure 7B. Cells were treated with DMSO or the indicated concentrations of losmapimod or C06 (1000, 200, and 40 nM). P-HSP27 (green, 800 nm channel) and total HSP27 (red, 700 nm channel) were detected by double blotting on the same membrane. Molecular weight marker corresponds to Precision Plus Protein Dual Color Standards (Bio-Rad, #1610374).

Supplementary Figure S6

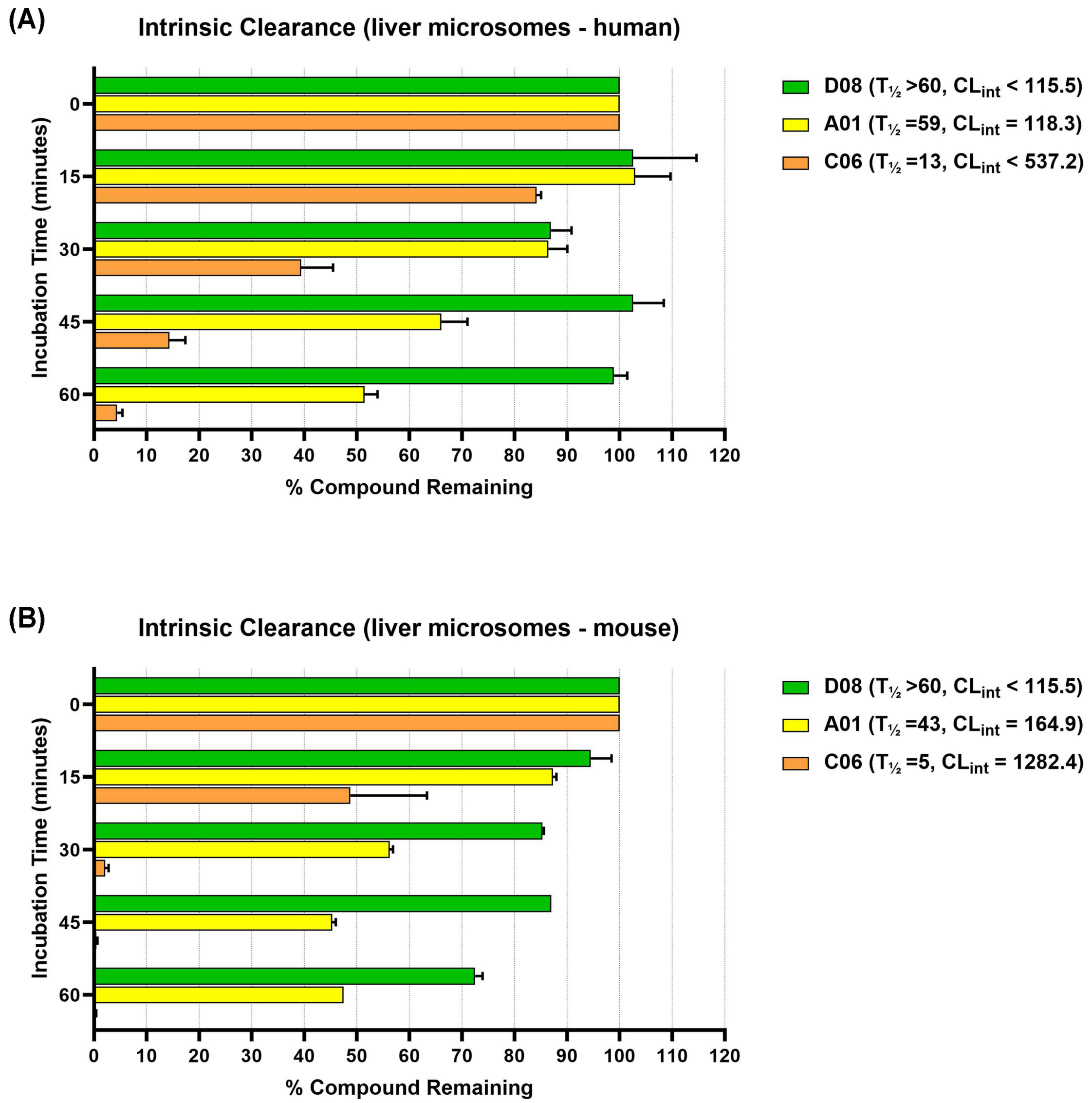

Supplementary Figure S6. ADME (absorption, distribution, metabolism, elimination) analysis.

Three compounds from the AI screen that effectively repress *DUX4-fl* and DUX4-FL targets (D08, A01, and C06) were tested for intrinsic clearance by liver microsomes using **(A)** human liver microsomes or **(B)** mouse liver microsomes. Data is shown as percent of the parent compound remaining at 15, 30, 45, and 60 minutes relative to that at the beginning. Data represent the mean of two experiments. The half-life ($T_{½}$) was estimated from the slope of the initial linear range of the logarithmic curve of compound remaining (%) vs. time, assuming first-order kinetics. The value of intrinsic clearance (CL_{int}, in µL/min/mg for microsomes) was calculated according to the following formula:

$$CL_{int} = \frac{0.693}{T_{1/2} * (\text{mg protein/µL or million cells/µL or pmol CYP isoyme/µL})}$$

Supplementary Table S1. Oligonucleotide primers

Primer name	Sequence
DUX4-fl-F (prePCR)	5′-GCTCTGCTGGAGGAGCTTTAGGA-3′
DUX4-fl-R (prePCR)	5′-CGCACTGCTCGCAGGTCTGCWGGT-3′
DUX4-fl-nested-F	5′-AGCTTTAGGACGCGGGGTTGGGAC-3′
DUX4-fl-nested-R	5′-GCAGGTCTGCWGGTACCTGG-3′
MBD3L2-F	5′-GCGTTCACCTCTTTTCCAAG-3′
MBD3L2-R	5′-GCCATGTGGATTTCTCGTTT-3′
TRIM43-F	5′-ACCCATCACTGGACTGGTGT-3′
TRIM43-R	5′-CACATCCTCAAAGAGCCTGA-3′
RPL13A-F	5′-AACCTCCTCCTTTTCCAAGC-3′
RPL13A-R	5′-GCAGTACCTGTTTAGCCACGA-3′

Supplementary Table S2. Kinome profiling results

Kinase Tested	Compound Concentration (μM)	Compound Name	% Inhibition		% Inhibition (mean)	Reference IC50 values		
			Point 1	Point 2		Staurosporine IC50 (μM)	SB202190 IC50 (μM)	Wortmannin IC50 (μM)
DDR2	0.5	C06	95	88	92	0.00061	1.58110	>10
EPH-A7	0.5	C06	89	90	90	0.19896	>10	>10
DDR1	0.5	C06	82	90	86	0.00119	0.35897	>10
EPH-A8	0.5	C06	81	89	85	0.10001	>10	>10
P38-ALPHA	0.5	C06	84	84	84	>10	0.00887	>10
EPH-A2	0.5	C06	81	81	81	0.27399	>10	>10
RET	0.5	C06	75	80	78	0.00120	>10	>10
EPH-B2	0.5	C06	69	85	77	0.03301	>10	>10
TIE2	0.5	C06	70	73	72	0.18741	>10	>10
RIPK1	0.5	C06	70	73	71	0.44282	>10	>10
BRAF	0.5	C06	72	68	70	0.00555	1.24514	>10
PDGFR-ALPHA	0.5	C06	67	68	67	<0.000128	>10	>10
FLT-3	0.5	C06	65	62	64	<0.000128	>10	>10
TRKC	0.5	C06	52	55	53	0.00091	>10	>10
P38-BETA	0.5	C06	54	51	53	>10	0.13076	>10
ARG	0.5	C06	45	59	52	0.07442	>10	>10
MUSK	0.5	C06	42	44	43	0.00067	>10	>10
EPH-B4	0.5	C06	31	50	41	1.01311	>10	>10
MNK2	0.5	C06	37	38	38	0.01453	>10	>10
PYK2	0.5	C06	38	37	37	0.01320	>10	>10
PDGFR-BETA	0.5	C06	32	42	37	<0.000128	>10	>10
CRAF	0.5	C06	33	33	33	>10	0.58838	>10
TRKB	0.5	C06	35	30	32	0.00094	>10	>10
EPH-B1	0.5	C06	30	33	32	0.35563	>10	>10
YES	0.5	C06	25	32	28	0.00312	>10	>10
EPH-A6	0.5	C06	7	49	28	0.03551	4.76853	>10
EPH-B3	0.5	C06	22	33	28	>10	>10	>10
BRK	0.5	C06	23	25	24	0.82074	3.16521	>10
TYK2	0.5	C06	25	20	23	<0.000128	>10	>10
HCK	0.5	C06	19	25	22	0.00148	>10	>10
FMS	0.5	C06	19	23	21	0.00022	>10	>10
LYNA	0.5	C06	15	27	21	0.00194	>10	>10
ATR	0.5	C06	17	25	21	>10	>10	3.43513
SRC	0.5	C06	15	27	21	0.00199	>10	>10
RSK3	0.5	C06	18	22	20	<0.000128	>10	>10
LOK	0.5	C06	26	14	20	<0.000128	>10	>10
FLT-1	0.5	C06	16	21	19	0.00800	>10	>10
MAP2K7	0.5	C06	38	-2	18	0.04783	>10	>10
FAK	0.5	C06	11	24	17	0.03764	>10	>10
PI3K-GAMMA	0.5	C06	9	25	17	9.97081	>10	0.00032
FGR	0.5	C06	29	5	17	0.00351	>10	>10
ABL1	0.5	C06	0	34	17	0.13721	>10	>10
TNIK	0.5	C06	16	15	16	0.00061	7.76309	>10
LYNB	0.5	C06	10	20	15	0.00293	>10	>10
KDR	0.5	C06	-5	35	15	0.00436	>10	>10
RON	0.5	C06	15	14	15	0.13811	>10	>10
KIT	0.5	C06	16	14	15	0.01189	>10	>10
EPH-A5	0.5	C06	-6	34	14	0.06542	>10	>10
FGFR1	0.5	C06	11	18	14	0.00793	>10	>10
EGFR	0.5	C06	20	8	14	1.31829	>10	>10
ERB-B4	0.5	C06	13	15	14	0.74760	>10	>10
FLT-4	0.5	C06	12	16	14	0.00105	>10	>10
FGFR4	0.5	C06	16	11	14	0.48975	>10	>10
JAK3	0.5	C06	7	20	14	<0.000128	>10	>10
MET	0.5	C06	17	10	13	0.37001	>10	>10

PI3K-ALPHA	0.5	C06	9	17	13	>10	>10	0.00025
CSK	0.5	C06	10	15	12	0.20783	>10	>10
EPH-A3	0.5	C06	-16	40	12	0.01759	>10	>10
SLK	0.5	C06	8	15	12	<0.000128	>10	>10
ITK	0.5	C06	12	11	12	0.00517	>10	>10
SRMS	0.5	C06	18	5	12	8.19732	>10	>10
TNK2	0.5	C06	12	11	11	0.00216	>10	>10
AKT2	0.5	C06	6	16	11	0.02658	>10	>10
P38-GAMMA	0.5	C06	16	6	11	0.59029	>10	>10
SYK	0.5	C06	9	13	11	0.00047	>10	>10
FGFR2	0.5	C06	12	9	11	0.00268	>10	>10
MAP4K5	0.5	C06	13	8	10	0.00014	>10	>10
MAP4K4	0.5	C06	17	3	10	<0.000128	>10	>10
TXK	0.5	C06	15	5	10	0.05646	>10	>10
AXL	0.5	C06	3	15	9	0.00149	>10	>10
IGF1R	0.5	C06	5	12	9	0.02636	>10	>10
TTK	0.5	C06	12	5	8	0.13024	>10	>10
ROS	0.5	C06	11	6	8	<0.000128	>10	>10
TYRO3	0.5	C06	12	4	8	0.00108	>10	>10
AKT1	0.5	C06	11	5	8	0.00971	>10	>10
TRKA	0.5	C06	9	6	8	0.00237	>10	>10
TNK1	0.5	C06	3	11	7	0.00041	>10	>10
MSK2	0.5	C06	15	-1	7	0.00383	>10	>10
LCK	0.5	C06	2	12	7	0.00247	>10	>10
ERB-B2	0.5	C06	11	2	7	0.57467	>10	>10
SGK2	0.5	C06	9	4	6	0.08926	>10	>10
TAK1-TAB1	0.5	C06	-10	23	6	0.03290	>10	>10
CLK1	0.5	C06	1	12	6	0.00275	>10	1.14316
SPHK1	0.5	C06	7	5	6	>10	>10	>10
MER	0.5	C06	5	7	6	0.07643	>10	>10
FGFR3	0.5	C06	7	4	6	0.41618	>10	>10
EPH-A1	0.5	C06	5	6	6	0.04744	>10	>10
TAOK2	0.5	C06	12	0	6	0.01079	>10	>10
TEC	0.5	C06	4	4	4	0.08132	>10	>10
LTK	0.5	C06	0	8	4	0.00233	>10	>10
FES	0.5	C06	-4	11	4	0.00238	>10	>10
IRR	0.5	C06	2	4	3	0.54753	>10	>10
ZAP70	0.5	C06	3	3	3	0.00218	>10	>10
RSK2	0.5	C06	0	6	3	<0.000128	>10	>10
LZK	0.5	C06	8	-2	3	0.68856	>10	>10
MST1	0.5	C06	-1	4	2	<0.000128	>10	>10
PKC-BETA1	0.5	C06	6	-3	2	<0.000128	1.37659	3.27885
ATM	0.5	C06	-5	8	2	>10	>10	0.03369
IRAK1	0.5	C06	4	-1	1	0.09995	>10	>10
JAK1	0.5	C06	2	0	1	0.00119	>10	>10
MAP4K1	0.5	C06	4	-2	1	<0.000128	>10	>10
PI3K-DELTA	0.5	C06	-9	10	0	>10	>10	0.00072
MRCKA	0.5	C06	0	0	0	0.00084	>10	>10
PKC-ALPHA	0.5	C06	-1	1	0	<0.000128	>10	3.31745
NEK4	0.5	C06	3	-5	-1	>10	>10	>10
P38-DELTA	0.5	C06	-4	1	-1	2.02836	>10	>10
SPHK2	0.5	C06	-9	7	-1	>10	>10	>10
CK1-DELTA	0.5	C06	-2	-1	-2	>10	0.16992	>10
PAK4	0.5	C06	-3	-1	-2	0.00511	>10	>10
PRKD3	0.5	C06	-5	1	-2	0.00091	>10	>10
MST2	0.5	C06	-4	-1	-2	0.00013	>10	>10
DYRK1B	0.5	C06	2	-7	-3	0.00471	>10	>10
RIPK2	0.5	C06	-3	-3	-3	0.44278	0.04377	>10
PDK1	0.5	C06	-3	-2	-3	0.00416	>10	>10
BMX	0.5	C06	-14	8	-3	0.00793	>10	>10
NEK3	0.5	C06	1	-7	-3	>10	>10	3.75978

PAK3	0.5	C06	-3	-3	-3	0.00126	>10	>10
ULK3	0.5	C06	-7	1	-3	0.00066	>10	>10
ALK	0.5	C06	-7	1	-3	0.00117	>10	>10
RSK4	0.5	C06	-2	-5	-3	<0.000128	>10	>10
SGK1	0.5	C06	-5	-1	-3	0.02682	>10	>10
PIM-1-KINASE	0.5	C06	0	-7	-4	0.00246	>10	0.21747
SSTK	0.5	C06	-4	-3	-4	>10	>10	>10
CDK5-P25	0.5	C06	-4		-4	0.00154	>10	>10
FYN	0.5	C06	-9	1	-4	0.00645	>10	>10
CK2	0.5	C06	-3	-5	-4	9.89638	>10	>10
P70S6K1	0.5	C06	-7	-3	-5	0.00159	>10	>10
PKC-GAMMA	0.5	C06	-7	-3	-5	<0.000128	>10	>10
CAMKK1	0.5	C06	-2	-8	-5	0.00465	>10	>10
ALK4	0.5	C06	2	-13	-6	>10	>10	>10
NEK6	0.5	C06	-2	-9	-6	>10	>10	>10
DAPK1	0.5	C06	-7	-4	-6	0.00260	>10	7.31074
HIPK4	0.5	C06	-4	-7	-6	0.22527	>10	1.30449
TLK1	0.5	C06	-8	-4	-6	0.02480	>10	>10
EIF2AK4	0.5	C06	-9	-3	-6	0.23459	>10	>10
PIM2	0.5	C06	-7	-5	-6	0.01671	>10	1.22732
CDK9-CYCLINT1	0.5	C06	-8	-4	-6	0.00521	>10	>10
CAMK2G	0.5	C06	-5	-6	-6	<0.000128	>10	>10
MAPKAPK-2	0.5	C06	-3	-9	-6	0.04190	>10	>10
CK1	0.5	C06	-6	-5	-6	9.18203	1.08448	>10
GAK	0.5	C06	-13	1	-6	0.00538	0.07231	4.17885
IRE1	0.5	C06	-7	-5	-6	0.09769	>10	>10
PASK	0.5	C06	-3	-10	-6	0.01843	>10	>10
BLK	0.5	C06	-18	6	-6	0.00235	>10	>10
NEK2	0.5	C06	-6	-6	-6	0.82949	>10	>10
ICK	0.5	C06	-5	-7	-6	0.00581	>10	>10
HIPK2	0.5	C06	-9	-4	-7	0.41543	>10	0.04962
GSK-3-ALPHA	0.5	C06	-6	-8	-7	0.00915	>10	2.51914
TSSK1	0.5	C06	-9	-4	-7	<0.000128	>10	>10
CLK4	0.5	C06	-14	0	-7	0.00228	>10	0.51890
PKC-EPSILON	0.5	C06	-10	-3	-7	0.00018	>10	>10
MYO3A	0.5	C06	-1	-13	-7	0.39527	>10	>10
PDK2	0.5	C06	-4	-10	-7	>10	>10	>10
MRCKB	0.5	C06	-10	-4	-7	0.00127	>10	>10
ALK5	0.5	C06	0	-14	-7	7.31590	>10	>10
DYRK2	0.5	C06	0	-15	-8	0.47149	>10	>10
NDR1	0.5	C06	-8	-8	-8	0.00050	>10	>10
DRAK1	0.5	C06	-7	-8	-8	0.01285	>10	>10
CAMK4	0.5	C06	-9	-7	-8	0.28639	>10	>10
SRPK1	0.5	C06	-6	-10	-8	0.72617	>10	>10
CAMK2D	0.5	C06	-11	-4	-8	<0.000128	>10	>10
MSK1	0.5	C06	-8	-8	-8	0.00034	>10	>10
MARK3	0.5	C06	-3	-14	-8	<0.000128	>10	>10
PDK4	0.5	C06	-4	-12	-8	>10	>10	>10
MARK4	0.5	C06	-7	-10	-9	<0.000128	>10	>10
TAOK3	0.5	C06	-9	-9	-9	0.00320	>10	>10
LATS1	0.5	C06	-3	-14	-9	0.00288	>10	>10
CDK5-P35	0.5	C06	-4	-13	-9	0.00308	>10	>10
NEK9	0.5	C06	-9	-9	-9	0.21784	>10	0.46735
PKC-IOTA	0.5	C06	-3	-15	-9	0.01836	>10	>10
PRKG2	0.5	C06	-9	-9	-9	<0.000128	9.46865	>10
SGK3	0.5	C06	-11	-7	-9	0.15634	>10	>10
MAP2K4	0.5	C06	-11	-7	-9	0.01019	>10	>10
BTK	0.5	C06	-16	-2	-9	0.11455	>10	>10
TSSK3	0.5	C06	-11	-8	-9	0.00855	>10	>10
NEK1	0.5	C06	-19	0	-10	0.04597	>10	>10

P70S6K2	0.5	C06	-12	-7	-10	0.00246	>10	>10
PLK4	0.5	C06	-15	-4	-10	0.00134	>10	>10
PAK2	0.5	C06	-11	-8	-10	0.00341	>10	>10
CK1-GAMMA3	0.5	C06	-9	-11	-10	5.19496	>10	1.07631
CRIK	0.5	C06	-8	-11	-10	0.06940	1.00146	>10
ULK1	0.5	C06	-12	-8	-10	0.00698	>10	>10
BUB1	0.5	C06	-12	-8	-10	0.50196	9.19314	>10
PAK5	0.5	C06	-12	-8	-10	0.00896	>10	>10
NEK5	0.5	C06	-9	-11	-10	0.05773	>10	1.75961
GSK-3-BETA	0.5	C06	4	-24	-10	0.00555	5.78888	0.81312
RSK1	0.5	C06	-8	-12	-10	0.00028	>10	>10
BRSK2	0.5	C06	-10	-11	-10	<0.000128	>10	>10
PKC-ZETA	0.5	C06	-9	-12	-11	0.10372	>10	>10
DYRK3	0.5	C06	-11	-11	-11	0.01709	>10	>10
PLK2	0.5	C06	-8	-14	-11	1.65830	>10	0.08119
CK1-GAMMA2	0.5	C06	-7	-14	-11	8.02594	>10	1.35833
PTK5	0.5	C06	-14	-8	-11	0.15230	>10	>10
DAPK3	0.5	C06	-10	-12	-11	0.00264	>10	>10
PRKG1	0.5	C06	-16	-7	-11	0.00104	>10	>10
CLK3	0.5	C06	-11	-12	-11	3.17111	>10	4.10123
FER	0.5	C06	-15	-8	-12	0.00510	>10	>10
ROCK1	0.5	C06	-10	-14	-12	0.00072	>10	>10
ALK2	0.5	C06	-11	-12	-12	1.94450	>10	>10
IKK-EPSILON	0.5	C06	-12	-12	-12	0.00091	>10	>10
CDK3-CYCLINE	0.5	C06	-12	-11	-12	0.00389	>10	>10
PIM3	0.5	C06	-17	-7	-12	<0.000128	>10	2.29479
TTBK2	0.5	C06	-14	-10	-12	>10	>10	>10
HIPK3	0.5	C06	-9	-15	-12	0.40118	>10	0.05176
INSR	0.5	C06	-15	-9	-12	0.02076	>10	>10
ALK6	0.5	C06	-15	-9	-12	>10	>10	>10
MSSK1	0.5	C06	-9	-16	-12	>10	>10	>10
LRRK2-G2019S	0.5	C06	-17	-8	-12	0.00015	>10	>10
STK25	0.5	C06	-15	-10	-12	0.00420	>10	>10
mTORC1	0.5	C06	-8	-17	-12	>10	>10	1.86057
IKK-BETA	0.5	C06	-11	-15	-13	1.00921	>10	>10
TBK1	0.5	C06	-24	-1	-13	0.00081	>10	>10
QIK	0.5	C06	-16	-9	-13	0.00075	>10	>10
MINK	0.5	C06	-12	-14	-13	0.00180	>10	>10
DYRK4	0.5	C06	-11	-15	-13	>10	>10	>10
AMPK-A2B2G1	0.5	C06	-13	-13	-13	<0.000128	>10	>10
PKAC-BETA	0.5	C06	-13	-13	-13	0.00093	3.35126	>10
IRAK4	0.5	C06	-7	-19	-13	0.00943	>10	>10
AURORA-A	0.5	C06	-14	-12	-13	0.00125	>10	>10
PBK	0.5	C06	-11	-16	-13	0.06670	>10	>10
PLK3	0.5	C06	-9	-18	-13	>10	>10	0.15947
DYRK1A	0.5	C06	-13	-13	-13	0.00570	>10	>10
PKN3	0.5	C06	-11	-16	-13	0.00015	0.06801	>10
CK1-EPSILON	0.5	C06	-13	-15	-14	4.33692	1.22347	>10
GRK3	0.5	C06	-13	-15	-14	0.80100	>10	>10
PDK3	0.5	C06	-14	-14	-14	>10	>10	>10
ROCK2	0.5	C06	-14	-14	-14	0.00032	>10	>10
DCAMKL1	0.5	C06	-17	-11	-14	0.52181	>10	>10
TESK1	0.5	C06	-13	-15	-14	0.26668	>10	>10
NIM1K	0.5	C06	-18	-11	-14	0.94607	>10	>10
PI4-K-BETA	0.5	C06	-15	-14	-15	>10	>10	0.05625
IKK-ALPHA	0.5	C06	-12	-17	-15	0.25709	>10	>10
MYO3B	0.5	C06	-16	-14	-15	0.16942	>10	>10
NUAK2	0.5	C06	-12	-18	-15	0.00050	>10	>10
AURORA-C	0.5	C06	-13	-17	-15	0.00129	>10	>10
HIPK1	0.5	C06	-12	-18	-15	0.93413	>10	0.09903

MELK	0.5	C06	-15	-15	-15	<0.000128	>10	2.64552
PRKD2	0.5	C06	-12	-20	-16	0.00126	>10	>10
CLK2	0.5	C06	-22	-9	-16	0.00527	>10	0.84633
AURORA-B	0.5	C06	-21	-10	-16	0.00051	>10	>10
PKAC-GAMMA	0.5	C06	-22	-10	-16	0.00377	0.62978	>10
MLK3	0.5	C06	-16		-16	0.00843	>10	>10
PI3K-BETA	0.5	C06	-15	-17	-16	>10	>10	0.00041
CK2A2	0.5	C06	-15	-17	-16	0.94840	>10	>10
MST3	0.5	C06	-14	-18	-16	0.00207	>10	>10
STK16	0.5	C06	-19	-14	-16	0.12130	>10	>10
TSSK2	0.5	C06	-10	-22	-16	0.00386	>10	>10
NDR2	0.5	C06	-15	-18	-16	0.00108	>10	>10
PRAK	0.5	C06	-17	-16	-16	0.52118	>10	>10
PKN2	0.5	C06	-14	-19	-17	0.00021	>10	>10
CAMK1D	0.5	C06	-12	-22	-17	0.00318	>10	>10
PAR1BA	0.5	C06	-18	-16	-17	<0.000128	>10	>10
CDK6-CYCLIND3	0.5	C06	-11	-23	-17	0.03231	>10	>10
PKA	0.5	C06	-13	-21	-17	0.00047	3.62142	>10
SRPK2	0.5	C06	-18	-16	-17	1.08807	>10	>10
PKC-ETA	0.5	C06	-17	-16	-17	<0.000128	>10	>10
PKC-THETA	0.5	C06	-16	-18	-17	<0.000128	>10	4.85311
MEKK3	0.5	C06	-17	-18	-17	0.00780	>10	>10
AMPK-A2B1G1	0.5	C06	-19	-16	-17	<0.000128	>10	>10
PKC-DELTA	0.5	C06	-9	-26	-18	<0.000128	>10	>10
CDK2-CYCLINE	0.5	C06	-17	-18	-18	0.00808	>10	>10
CDK1-CYCLINB	0.5	C06	-14	-22	-18	0.00346	>10	>10
AMPK-A1B1G1	0.5	C06	-10	-27	-18	<0.000128	>10	>10
CAMK2A	0.5	C06	-13	-23	-18	<0.000128	>10	>10
PHK-GAMMA1	0.5	C06	-19	-18	-18	<0.000128	>10	>10
PKN1	0.5	C06	-19	-18	-18	0.00013	>10	>10
MAPK1	0.5	C06	-19	-18	-18	3.37047	>10	>10
EIF2AK2	0.5	C06	-19	-18	-19	0.06776	>10	>10
PHK-GAMMA2	0.5	C06	-20	-17	-19	0.00013	>10	>10
CHEK2	0.5	C06	-21	-16	-19	0.01990	>10	>10
CDK4-CYCLIND	0.5	C06	-17	-20	-19	0.02734	>10	>10
MLK2	0.5	C06	-18	-20	-19	0.00529	3.13215	>10
EIF2AK1	0.5	C06	-13	-25	-19	6.88377	>10	>10
LATS2	0.5	C06	-14	-24	-19	0.00061	>10	>10
CAMK1A	0.5	C06	-14	-24	-19	0.07760	>10	>10
CK1-GAMMA1	0.5	C06	-16	-23	-20	>10	>10	1.36284
AKT3	0.5	C06	-17	-23	-20	0.00669	>10	>10
SIK	0.5	C06	-18	-23	-20	0.00035	>10	>10
MAP4K2	0.5	C06	-19	-21	-20	0.00022	>10	>10
PRKD1	0.5	C06	-18	-24	-21	0.00188	>10	>10
MEK2	0.5	C06	-21	-21	-21	0.00042	>10	>10
MAPK3	0.5	C06	-25	-16	-21	3.15115	>10	>10
GRK7	0.5	C06	-29	-13	-21	0.00845	>10	>10
PAK1	0.5	C06	-21	-21	-21	0.00141	>10	>10
DGKz	0.5	C06	-18	-25	-21	>10	>10	>10
ASK1	0.5	C06	-23	-20	-22	0.01159	>10	>10
JNK2	0.5	C06	-21	-23	-22	6.19642	>10	>10
PLK1	0.5	C06	-20	-23	-22	1.76491	>10	0.03620
MAPKAPK-3	0.5	C06	-26	-18	-22	3.26445	>10	>10
JNK1	0.5	C06	-21	-23	-22	5.71654	>10	>10
MAP3K8	0.5	C06	-31	-13	-22	>10	>10	>10
MST4	0.5	C06	-22	-24	-23	0.00324	>10	>10
MEK1	0.5	C06	-24	-22	-23	0.00073	>10	>10

STK33	0.5	C06	-23	-23	-23	0.00703	>10	>10
GRK5	0.5	C06	-21	-27	-24	0.03188	>10	>10
NEK7	0.5	C06	-25	-23	-24	>10	>10	>10
DCAMKL2	0.5	C06	-26	-23	-25	0.00867	>10	>10
PKC-BETA2	0.5	C06	-28	-22	-25	0.00053	>10	>10
ARK5	0.5	C06	-19	-32	-25	0.00050	>10	>10
PAK6	0.5	C06	-17	-34	-25	0.00249	>10	>10
PRKX	0.5	C06	-23	-27	-25	0.00076	>10	>10
BMPR2	0.5	C06	-25	-27	-26	>10	>10	>10
JAK2	0.5	C06	-22	-29	-26	0.00015	>10	>10
DRAK2	0.5	C06	-39	-13	-26	0.01250	>10	>10
LIMK1	0.5	C06	-23	-32	-27	0.00243	>10	>10
DLK	0.5	C06	-21	-33	-27	1.83133	>10	>10
CAMK2B	0.5	C06	-28	-27	-27	0.00042	>10	>10
MAP4K3	0.5	C06	-16	-40	-28	<0.000128	>10	>10
TTBK1	0.5	C06	-29	-28	-28	>10	>10	>10
MNK1	0.5	C06	-28	-31	-30	0.06993	>10	>10
CHEK1	0.5	C06	-30	-29	-30	<0.000128	>10	>10
AMPK-A1B2G1	0.5	C06	-31	-29	-30	<0.000128	>10	>10
CDK2-CYCLINA	0.5	C06	-31	-29	-30	0.00222	>10	>10
JNK3	0.5	C06	-32	-28	-30	2.82612	>10	>10
DGKa	0.5	C06	-25	-37	-31	>10	>10	>10
mTOR	0.5	C06	-40	-26	-33	1.10726	>10	1.15961
MARK1	0.5	C06	-32	-34	-33	<0.000128	>10	>10
PIKFYVE	0.5	C06	-32	-36	-34	0.09161	0.01880	3.46847
EIF2AK3	0.5	C06	-37	-32	-34	>10	>10	>10
GRK6	0.5	C06	-40	-30	-35	0.04276	>10	>10
MLK1	0.5	C06	-46	-28	-37	0.00023	5.61304	>10
MEK3	0.5	C06	-39	-38	-38	0.05359	>10	>10
WEE1	0.5	C06	-36	-42	-39	3.40721	>10	>10
HASPIN	0.5	C06	-41	-42	-41	0.01084	>10	>10
EPH-A4	0.5	C06	-39	-46	-42	0.89313	>10	>10
BRSK1	0.5	C06	-42	-44	-43	0.00036	>10	>10
WNK1	0.5	C06	-42	-49	-45	>10	>10	>10
DGKb	0.5	C06	-41	-52	-46	>10	>10	>10
CDK7	0.5	C06	-46	-49	-48	0.01884	>10	>10
LKB1	0.5	C06	-34	-69	-52	0.00019	>10	>10

*empty well: knocked out data point

Supplementary Table S3. In vitro absorption study

Test Compound	MCULE I.D.	Test Concentration	Permeability (10^{-6} cm/s) 1st	2nd	Mean	Percent Recovery(%) 1st	2nd	Mean
**A-B permeability (Caco-2, pH 6.5/7.4)**								
D08	MCULE-8132215280	1.0E-05 M	0.16	0.17	0.2	74	89	82
A01	MCULE-6746692707	1.0E-05 M	5.99	6.29	6.1	79	78	78
C06	MCULE-2965976181	1.0E-05 M	0.50	0.53	0.5	24	25	25
**B-A permeability (Caco-2, pH 6.5/7.4)**								
D08	MCULE-8132215280	1.0E-05 M	0.45	0.50	0.5	111	103	107
A01	MCULE-6746692707	1.0E-05 M	0.21	0.27	0.2	20	36	28
C06	MCULE-2965976181	1.0E-05 M	5.38	4.52	4.9	17	20	19

The apparent permeability coefficent (P_{app}) of the test compound was calculated as follows:

$$P_{app}(cm/s) = \frac{V_R * C_{R,end}}{\Delta t} * \frac{1}{A * (C_{D,mid} - C_{R,mid})}$$

where V_R is the volume of the receiver chamber. $C_{R,end}$ is the concentration of the test compound in the receiver chamber at the end time point, Δt is the incubation time, and A is the surface area of the cell monolayer. $C_{D,mid}$ is the calculated mid-point concentration of the test compound in the donor side, which is the mean value of the donor concentration at time 0 minute and the donor concentration at the end time point. $C_{R,mid}$ is the mid-point concentration of the test compound in the receiver side, which is one half of the receiver concentration at the end time point. Concentrations of the test compound were expressed as peak areas of the test compound.

The recovery of the test compound was calculated as follows:

$$Recovery(\%) = \frac{V_D * C_{D,end} + V_R * C_{R,end}}{V_D * C_{D0}} * 100$$

where V_D and V_R are the volumes of the donor and receiver chambers, respectively. $C_{D,end}$ is the concentration of the test compound in the donor sample at the end time point. $C_{R,end}$ is the concentration of the test compound in the receiver sample at the end time point. C_{D0} is the concentration of the test compound in the donor sample at time zero. Concentrations of the test compound are expressed as peak areas of the test compound.

References

1 Han, S. *et al.* Structural Characterization of Proline-rich Tyrosine Kinase 2 (PYK2) Reveals a Unique (DFG-out) Conformation and Enables Inhibitor Design. *J Biol Chem* **284**, 13193–13201, doi:10.1074/jbc.M809038200 (2009).
